# Cohort study of medical cannabis authorization and motor vehicle crash-related healthcare visits in 2014–2017 in Ontario, Canada

**DOI:** 10.1186/s40621-021-00321-1

**Published:** 2021-04-28

**Authors:** Cerina Lee, Don Voaklander, Jasjeet K. Minhas-Sandhu, John G. Hanlon, Elaine Hyshka, Jason R. B. Dyck, Dean T. Eurich

**Affiliations:** 1 School of Public Health, University of Alberta, 2-040 Li Ka Shing Centre for Health Research Innovation 11,203–87 Avenue, Edmonton, Alberta AB T6G 2E1 Canada; 2 St. Michael’s Hospital Department of Anesthesia, University of Toronto, Toronto, Ontario Canada; 3 Department of Anaesthesiology and Pain Medicine, University of Toronto, Toronto, Ontario Canada; 4 Cardiovascular Research Centre, Department of Pediatrics, Faculty of Medicine and Dentistry, University of Alberta, Edmonton, Alberta Canada

**Keywords:** Medical cannabis, Motor vehicle crash, Healthcare utilization, Public health

## Abstract

**Background:**

With increasing numbers of countries/jurisdictions legalizing cannabis, cannabis impaired driving has become a serious public health concern. Despite substantive research linking cannabis use with higher rates of motor vehicle crashes (MVC), there is an absence of conclusive evidence linking MVC risk with medical cannabis use. In fact, there is no clear understanding of the impact of medical cannabis use on short- and long-term motor vehicle-related healthcare visits. This study assesses the impact of medical cannabis authorization on motor vehicle-related health utilization visits (hospitalizations, ambulatory care, emergency department visits, etc) between 2014 and 2017 in Ontario, Canada.

**Methods:**

A matched cohort study was conducted on patients authorized to use medical cannabis and controls who did not receive authorization for medical cannabis – in Ontario, Canada. Overall, 29,153 adult patients were identified and subsequently linked to the administrative databases of the Ontario Ministry of Health, providing up to at least 6 months of longitudinal follow-up data following the initial medical cannabis consultation. Interrupted time series analyses was conducted to evaluate the change in rates of healthcare utilization as a result of MVC 6 months before and 6 months after medical cannabis authorization.

**Results:**

Over the 6-month follow-up period, MVC-related visits in medical cannabis patients were 0.50 visits/10000 patients (*p* = 0.61) and − 0.31 visits/10000 patients (*p* = 0.64) for MVC-related visits in controls. Overall, authorization for medical cannabis was associated with an immediate decrease in MVC-related visits of − 2.42 visits/10000 patients (*p* = 0.014) followed by a statistically significant increased rate of MVC-related visits (+ 0.89 events/10,000 in those authorized medical cannabis) relative to controls in the period following their authorization(*p* = 0.0019). Overall, after accounting for both the immediate and trend effects, authorization for medical cannabis was associated with an increase of 2.92 events/10,000 (95%CI 0.64 to 5.19) over the entire follow-up period. This effect was largely driven by MVC-related emergency department visits (+ 0.80 events/10,000, *p* < 0.001).

**Conclusions:**

Overall, there was an association between medical cannabis authorization and healthcare utilization, at the population level, in Ontario, Canada. These findings have public health importance and patients and clinicians should be fully educated on the potential risks. Continued follow-up of medically authorized cannabis patients is warranted to fully comprehend long-term impact on motor vehicle crash risk.

## Background

Since 2001, Canadians have been allowed to legally possess cannabis for medical purposes with a health care provider’s authorization(Alberta, 2017). With non-medical cannabis legalization in Canada and certain states in the United States, there is rising public concern about cannabis-impaired driving/driving under the influence of cannabis (DUIC) (Valleriani, 2017). Past fatality studies (Andrews et al., 2015; Callaghan et al., 2013; Fischer et al., 2016; Romano et al., 2017) resulting from motor vehicle crashes (MVC) suggest higher risk of MVC is associated with general cannabis consumption, however, there is a lack of robust evidence (Rogeberg and Elvik, 2016) surrounding MVC risk for medical cannabis users at the population level.

Previous research on cannabis use and MVC risk have shown mixed results -with a continued debate in the literature on whether or not this association is significant. Clinical studies have reported common physiological effects (both acute and long-term exposure of cannabis use) on the brain that have been found to impair driving ability (Neavyn et al., 2014; Ogourtsova et al., 2018; Wright and Metts, 2016). Evidence suggests that the risk of being involved in a motor vehicle crash increases approximately two-fold when a person drives immediately after smoking cannabis (Hartman and Huestis, 2013) and that acute cannabis intoxication may be associated with an increased MVC risk (Rogeberg and Elvik, 2016). In Canada, statistical data has shown that the percentage of fatally injured drivers from testing positive for cannabis, has generally increased over time (Foundation, T. I. R, 2018). Conversely, higher levels of tetrahydrocannabinol (THC) in the blood has been correlated with higher rates of MVC and impaired driving behaviors, but not at lower levels (Brubacher et al., 2019; Bonar et al., 2019). Further, other studies have shown a nonsignificant association between traffic accidents and cannabis use (Hostiuc et al., 2018; Hansen et al., 2018). In other jurisdictions where medical cannabis has been legalized (i.e. Colorado), an increased rate of MVCs has been reported; whereas the rate remained the same in states without cannabis legalization (Salomonsen-Sautel et al., 2014).

To address the evidence gap, research is needed on whether medical use of cannabis is associated with a higher risk of MVC. Although cannabis would be expected to have a similar potential for MVC in these patients, our study examines whether these medical cannabis patients represent a different subset of the cannabis using population with potentially different patterns of risk behaviors. While past studies on causal interpretation between medical cannabis impairment and motor vehicle crashes present mixed results - a majority of cohort studies are limited due to small sample sizes (Bonar et al., 2019; Ogourtsova et al., 2018), are outdated (Walsh and Mann, 1999; Asbridge et al., 2005), express high publication bias (Hostiuc et al., 2018); do not differentiate between medical and recreational cannabis (Azofeifa et al., 2015; Li et al., 2012; Masten and Guenzburger, 2014), rely heavily on self-reported measures (Richer and Bergeron, 2009), and have loss of participants to follow up over time (Callaghan et al., 2013) who are using medical cannabis.

Thus, we conducted a large cohort study of adults authorized to obtain medical cannabis - to assess whether medical cannabis use has any association on healthcare utilization due to MVC. In this paper, we hypothesized that there is an association between medical authorization for cannabis and MVC-related healthcare utilization in comparison to controls.

## Methods

### Study design

A matched cohort study was conducted on patients authorized to use medical cannabis and controls who did not receive authorization for medical cannabis – in Ontario, Canada. This retrospective longitudinal matched cohort study is part of a larger study assessing the health outcomes of medical cannabis among patients who received medical authorization (Eurich et al., 2020).

### Study population

#### Inclusion Criteria

All adult patients authorized for medical cannabis [inhaled (smoked or vaporized) or orally consumed (oils) cannabis] that attended specialized cannabis clinics in Ontario (Canada) between April 24, 2014 and March 31, 2017. These individuals were ≥ 18 years of age, of any sex and ethnicity, and had received medical cannabis authorization for a variety of acute and chronic health conditions. Patients may choose to seek assessment for medical cannabis through the clinic via a self-referral or by a physician referral. The index date for each patient was the first recorded date of medical cannabis authorization at the clinics (Table 1).

**Table 1:** Characteristics of patients with six months follow-up before and six months after the index date included in interrupted time series analyses analysis (*n* = 27657^a^)

Characteristic	Unauthorized for medical cannabis (***N*** = 17,732)	Authorized for medical cannabis (***N*** = 9925)	***p***-value
Age			
< 21	143 (0.8%)	78 (0.8%)	0.9957
21 to 30	1855 (10.5%)	1063 (10.7%)	
31 to 40	3553 (20.0%)	1993 (20.1%)	
41 to 50	3876 (21.9%)	2135 (21.5%)	
to 60	4545 (25.6%)	2562 (25.8%)	
61 to 70	2527 (14.3%)	1414 (14.3%)	
71 to 80	891 (5.0%)	491 (5.0%)	
> 80	342 (1.9%)	189 (1.9%)	
Sex			
Female	8054 (45.4%)	4462 (45.0%)	0.4576
Male	9678 (54.6%)	5463 (55.0%)	
Nearest Census based neighborhood income quintile			
1	3963 (22.4%)	2212 (22.3%)	0.9939
2	3785 (21.4%)	2103 (21.2%)	
3	3347 (18.9%)	1893 (19.1%)	
4	3490 (19.7%)	1959 (19.7%)	
5	3147 (17.8%)	1758 (17.7%)	
Rural	1891 (10.7%)	797 (8.0%)	< 0.0001
Diagnosis codes			
Diabetes	1945 (11.0%)	1132 (11.4%)	0.2680
Congestive heart failure	97 (0.6%)	64 (0.6%)	0.3051
COPD	2028 (11.4%)	1187 (12.0%)	0.1933
Asthma	3438 (19.4%)	1965 (19.8%)	0.4096
Cancer	1250 (7.1%)	726 (7.3%)	0.4110
Musculoskeletal issues	7791 (43.9%)	4377 (44.1%)	0.7931
Neurologic disorders	2564 (14.5%)	1515 (15.3%)	0.0702
Pain	401 (2.3%)	280 (2.8%)	0.0040
Behavioural issues	3313 (18.7%)	1929 (19.4%)	0.1259
Fatigue	188 (1.1%)	139 (1.4%)	0.0120
Metabolic disease	2132 (12.0%)	1286 (13%)	0.0236
Anxiety at baseline	4313 (24.3%)	4867 (49.0%)	< 0.0001

^a^29153 adult patients were identified and subsequently linked to the administrative databases of the Ontario Ministry of Health providing up to at least 6 months of longitudinal follow-up data following the initial medical cannabis consultation. All data was released as de-identified data

*COPD* Chronic obstructive pulmonary disease

#### Exclusion Criteria

Adult patients who received medical cannabis authorization but were unable to be matched with at least one control, those who were non-eligible to Ontario Health Insurance Plan at baseline and those with invalid or duplicate identifiers were excluded. Patients who had less than 6 months administrative data before the index date and less than 6 months after, were also excluded. This restriction was to ensure we had sufficient health data to determine trends in health care utilization. Further, through sensitivity analysis, we excluded patients having less than 12 months data before the index date and less than 12 months data after.

#### Matched Controls

Each authorized medical cannabis patient was matched at the time of the case index up to 3 controls based on age (± 1 years), sex, Local Health Integration Network location, income quartile, and history of diabetes, heart disease, chronic obstructive pulmonary disease, asthma, cancer, musculoskeletal issues, neurological issues, pain, behavioral issues, fatigue, malnutrition, and metabolic disease based on any related ICD-9/10 codes within the previous 5 years. Matching was completed with replacement and thus an unauthorized patient could have been utilized for 1 or more authorized patients, although no controls was selected more than once. To be considered as unauthorized, no record of a referral to a participating cannabis clinic was allowed. After matching, a pseudo-index date equal to the authorized patient was assigned so that the distribution of index dates is the same as the authorized patients.

### Data source

All data for both cannabis users and matched controls were obtained from the provincial administrative health databases collected and housed by Ontario’s Institute for Clinical Evaluative Sciences. The ICES Data Repository consists of record-level, coded and linkable health data sets. It encompasses publicly funded administrative health services records for the Ontario population eligible for universal health coverage.

All adult patients seeking assessment at specialized cannabis clinics (between April 2014–March 2017) in Ontario, Canada were eligible. Informed consent was provided by the patient at the time of first intake, which allows data to be collected and used for clinical and research purposes. As part of the authorization and intake process, each patient seeking medical cannabis meets with a trained counselor who performs and initial assessment and collects relevant data. All patients must provide sociodemographic information and disclose their primary medical complaints that constitute their rationale for requesting a medical cannabis authorization. Following their initial intake interview, the patient is referred to a physician who makes their assessment based on the self-reported information, the patient’s health record, and any additional assessments conducted by the physician. Initial referral to the clinics can be a self-referral by the patients or by a medical professional.

Overall, 29,153 adult patients were identified and subsequently linked to the administrative databases of the Ontario Ministry of Health hospitalizations and emergency department visits providing up to at least 6 months of longitudinal follow-up data following the initial medical cannabis consultation. These data were provided by the ICES administrative databases in Ontario and all data was released as de-identified data. Research ethics approval was obtained from the University of Alberta Health Research Ethics Board (PRO 00083651) and Veritas Research Ethics Board (Ontario) (16111–13:21:103–01-2017).

### Outcomes

All types of healthcare resources utilization that was related or potentially due to motor vehicle crashes were considered in this study (hospitalizations or emergency department visits). The combined endpoint of MCV-related hospitalizations or emergency department was our variable of interest. For this endpoint, if a patient had an emergency department visit that directly lead to a hospitalization only 1 event was counted in the model. For the individual assessments of MVC-related hospitalization or emergency department visits, each was considered as mutually exclusive for analyses. This included ICD-10 codes V40-V69 (Appendix 1); MVC related to buses were not included (V70-V79).

### Study sample

**Fig. 1:**
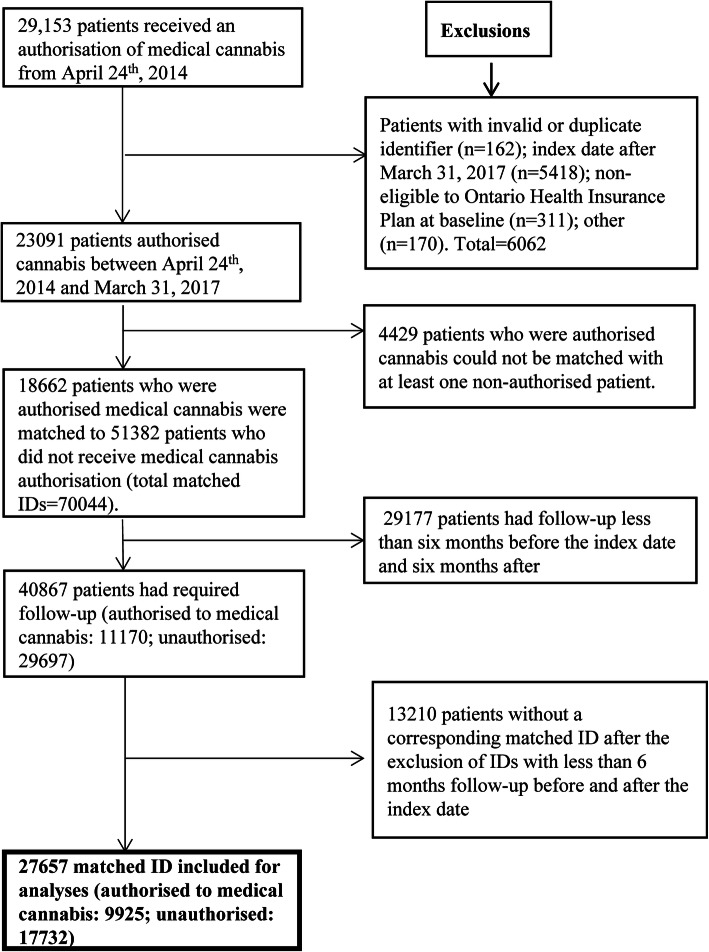
Selection of study population

In total, 29,153 patients attended a cannabis clinic and provided consent. Of these patients, 9925 medically authorized cannabis patients having at least 6 months follow-up data before and after the index date were matched to 17,732 controls (Fig. 1). In each group, at least 2/3 of the patients were aged 60 years or less, and the majority were men (55%). Musculoskeletal issues, anxiety, neurologic disorders, and asthma were the most predominant morbidities. Morbidities were well balanced between the two groups due to the matched study design although slightly fewer patients authorized for medical cannabis resided in a rural area (8% vs 10.7%) and were more likely to have a history of anxiety (49% vs 24.3%) (*p* < 0.001 for each).

### Statistical analysis

All data are expressed descriptively using means (standard deviations) or proportions as appropriate. To assess the effect of medical cannabis use on motor vehicle-related visits, interrupted time series (ITS) analyses assessed the trend in MVC in the 6 months before and 6 months after the authorization of cannabis (Wagner et al., 2002). Each outcome was assessed in 30-day windows for each patient (i.e., total number of occurrences in the month) which represents the time series before and after the change point (i.e. authorization for medical cannabis). Two parameters defined the time series – a level (immediate change in y-intercept) and trend (change in slope over time). The model accounts for the pretreatment trend differences between those authorized medical cannabis and controls. First the number of motor vehicle-related visits within each 30-day window are summated for the controls and medically authorized cannabis users separately. Then, the difference in motor vehicle-related visit outcomes between authorized and unauthorized patients is modeled using the standard controlled ITS approach (Zhang et al., 2009). The average pretreatment effect is then projected into the posttreatment period as the best estimate of the counterfactual—what motor vehicle-related visits would have been in the absence of authorization for medical cannabis (Linden, 2015; Bernal et al., 2017).

By modeling the outcomes in this manner, a clear interpretation of effects can be observed: the trend in those authorized for medical cannabis; the trend in those not authorized; and the joint trend of those authorized relative to those unauthorized; as opposed by just relative effects between authorized and those unauthorized where the true drivers of any differences may be difficult to interpret. In addition, the overall absolute effects of medical cannabis authorization on MVC was calculated, which summarizes both the immediate level change (i.e., within a month) and change in trend over the 6 months with the multivariate delta method used to the construct 95% confidence intervals around the estimate.

### Sensitivity analysis

To assess the effect of longer exposure to medical cannabis on motor vehicle-related visits, we extended the follow-up to 12 months before the index date and after exposure by repeating the ITS analysis for all outcomes. However, it is important to note that this additional extension period led to the exclusion of patients who did not have sufficient data 12 months prior or 12 months after (or in the matched controls). As the number of patients included in this analysis was significantly smaller, we considered this as an exploratory analysis.

In addition, we conducted a sensitivity analysis to exclude 0.1 and 0.6, as these codes relate to passengers. Patients involved in motor vehicle collisions involving cannabis and other substances sometimes indicate that they were a passenger as opposed to a driver to avoid any repercussion for the accident from law enforcement. As such, we elected to include all passenger codes in the main analysis.

## Results

**Fig. 2:**
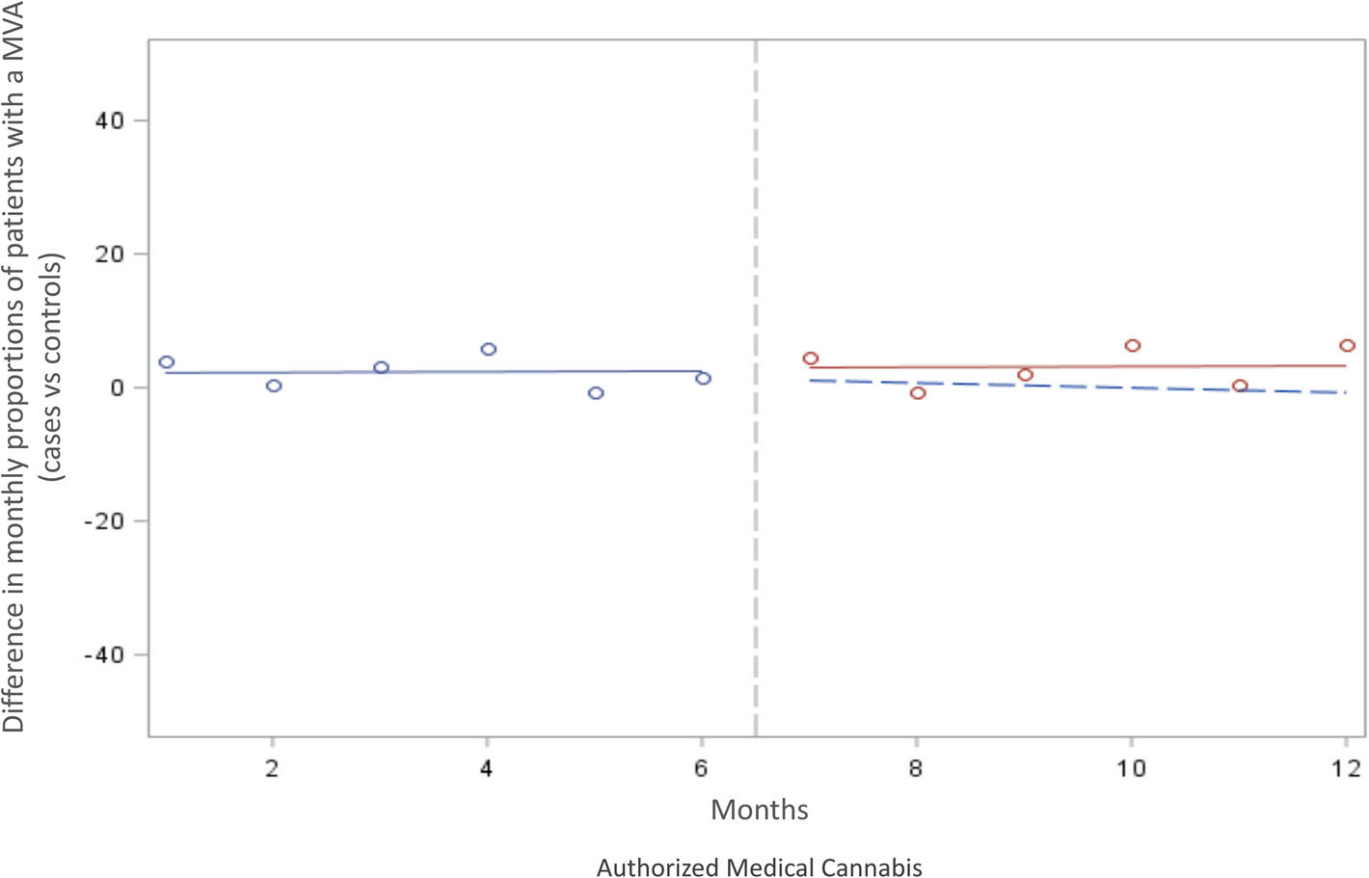
Interrupted Time Series Analyses: Difference in motor vehicle-related health care utilization by patients after authorization of medical cannabis compared to those unauthorized (*n* = 27,657). Legend:. *Healthcare utilization includes all hospitalizations and hospital visits. *Solid lines represent the pre trend (blue) and post trends (red) after authorization for medical cannabis. *Dashed line (blue) represents the counterfactual trend expected if no change occurred due to medical cannabis authorization

In the 6 months before authorization, there were 46 MVC-related health care visits/admissions per 10,000 patients among those authorized for medical cannabis and 32 MVC-related health care visits/admissions per 10,000 patients among those not authorized for medical cannabis (Table 2). Following medical cannabis authorization, an immediate (level change) change of − 3.15 MVC-related health care visits/admissions per 10,000 patients occurred whereas in controls − 2.38 MVC-related health care visits/admissions per 10,000 patients occurred (Neither change was statistically significant (*p* = 0.39 and *p* = 0.29, respectively). Furthermore, with respect to changes in trend, amoung those authorized for medical cannabis, MVC-related visits after 6 months was 0.50 visits per 10,000 patients; and MVC-related visits in controls was − 0.31 visits per 10,000 patients. Neither change was statistically significant (*p* = 0.61 and *p* = 0.64, respectively) (Table 3); and also shown by the ITS analysis in the difference in monthly proportions of healthcare utilization between cases and controls (Fig. 2). When evaluating the difference in events amoung those authorized medical cannabis to controls, an immediate decrease in MVC -related visits of − 2.42 events per 10,000 in those authorized medical cannabis was observed (level change) *p* = 0.0138). This was followed by an increase of MVC-related visits of 0.89 events per 10,000 in those authorized medical cannabis (over the 6 months relative to controls – trend change), which was statistically significant (*p* = 0.0019) (Table 3). After accounting for both the immediate (level) and temporal (trend) effects, authorization of medical cannabis was associated with an absolute increase of 2.92 events/10,000 (95%CI 0.64 to 5.19) over the entire follow-up period.

**Table 2:** Cannabis motor vehicle crash healthcare utilization – six months before and six months after authorization for medical cannabis

Outcome	Cannabis Population Difference in mean number of visits/admissions per 10,000 patients from 6 months before to 6 months after medical cannabis			Matched Controls Difference in mean number of visits/admissions per 10,000 patients from 6 months prior to 6 months after index date		
	Before	After	Change	Before	After	Change
Hospitalization or Emergency Department visit as a result of motor vehicle crashes	46	48	+ 2	32	34	+ 2
Hospitalization visit as a result of motor vehicle crashes	6.05	2.02	−4.03	2.82	0.56	−2.26
Emergency Department visit as a result of motor vehicle crashes	40	46	+ 6	29	33	+ 4

**Table 3:** Interrupted time series analysis of healthcare utilization due to motor vehicle crash six months before and six months after authorization of medical cannabis compared to those unauthorized (*n* = 27,657)

Outcome	Authorized Medical Cannabis				Unauthorized Controls				Difference			
	Immediate Level Change*		Temporal Trend change**		Immediate Level Change*		Temporal Trend Change**		Immediate Level Change*		Temporal Trend Change**	
	Events/ 10,000 patients	*p*-value	Events/ 10,000 patients	*p*-value	Events/10,000 patients	*p*-value	Events/ 10,000 patients	*p*-value	Events/ 10,000 patients	*p*-value	Events/ 10,000 patients	*P*-value
Motor-vehicle related hospitalization or ED visit	−3.15	0.3988	0.50	0.61	−2.38	0.2879	−0.31	0.64	−2.42	0.0138	0.89	0.0019
Absolute Effect Motor-vehicle related hospitalization or ED visit									**Events/10,000 patients** 2.92		**95% Confidence Intervals** 0.64–5.19	
Motor-vehicle related hospitalization	−1.97	0.0365	0.22	0.2753	−0.91	0.0749	−0.068	0.5782	−1.10	0.7322	−0.0081	0.9898
Absolute Effect Motor-vehicle related hospitalization									**Events/10,000 patients** − 1.15		**95% Confidence Intervals** (− 14.63–12.33)	
Motor-vehicle related ED	− 1.91	0.5108	0.64	0.4184	−1.42	0.4661	−0.18	0.7384	−0.90	0.2907	0.80	0.0001
Absolute Effect Motor-vehicle related ED visit									**Events/10,000 patients** 3.92		**95% Confidence Intervals** (2.65–5.19)	

*change in the month following the authorization of cannabis or the index date

**change in slope in the six months following the authorization of cannabis or the index date

*ED* Emergency department

Stratified analyses by type of MVC-related visit suggests that emergency department visits contributed to the majority of the difference observed between those authorized medical-cannabis compared to controls. Indeed, although no statistical difference was observed with respect to MVC-related hospitalizations immediately (level change) or during the follow-up (trend change) or immediately in MVC-related emergency department visits (level change), an increase of MVC-related emergency department visits was observed of 0.80 events per 10,000 in those authorized medical cannabis during the follow-up (over the 6 months relative to controls; trend change, *p* = 0.0001) No clinically important differences were noted for either age or sex (Appendix 2 and 3).

### Additional sensitivity analyses

After exclusion of 0.1 and 0.6 (codes relating to passengers), following medical cannabis authorization, MVC-related visits in medical cannabis patients after 6 months was 0.46 visits per 10,000 patients; and MVC-related visits in controls was − 0.57 visits per 10,000 patients - with neither change statistically significant (*p* = 0.54 and *p* = 0.32, respectively) (Table 4). After accounting for both the immediate and temporal effects, the absolute effect of medical cannabis authorization was a non-statistcially significant increase of 2.34 events/10,000 (95%CI: − 25.06-29.74) over the 6-month follow-up period.

**Table 4:** Sensitivity Analyses (Exclusion of 0.1 & 0.6 Codes): Interrupted time series analysis of healthcare utilization due to motor vehicle crash six months before and six months after authorization of medical cannabis compared to those unauthorized (*n* = 27,657)

Outcome	Authorized Medical Cannabis				Unauthorized Controls				Difference				Absolute Difference	
	Immediate Level Change*		Temporal Trend change**		Immediate Level Change*		Temporal Trend Change**		Immediate Level Change*		Temporal Trend Change**		Combined Level and Trend Changes	
	Events/ 10,000 patients	*p*-value	Events/ 10,000 patients	*p*-value	Events/ 10,000 patients	*p*-value	Events/ 10,000 patients	*p*-value	Events/ 10,000 patients	*p*-value	Events/ 10,000 patients	*P*-value	Events/ 10,000 patients	95% Confidence intervals
Motor-vehicle related hospitalization or ED visit	−2.50	0.3745	0.46	0.5358	−0.23	0.9196	−0.57	0.3239	−0.95	0.9229	0.55	0.8284	2.34	−25.06 to 29.7
Motor-vehicle related hospitalization	−1.56	0.0857	0.08	0.7079	−0.64	0.0586	−0.0063	0.9369	−0.65	0.4911	0.28	0.3066	1.02	−2.96 to 4.99
Motor-vehicle related ED	−0.85	0.7276	0.39	0.5517	0.53	0.8388	−0.63	0.2613	3.20	0.2904	0.35	0.7655	5.29	−6.60 to 17.17

When we extended our analysis out to 12 months, in the 12 months before authorization, there were 121 MVC-related health care visits/admissions per 10,000 patients among those authorized medical cannabis and 65 MVC-related health care visits/admissions per 10,000 patients among those not authorized medical cannabis (Table 5). Following medical cannabis authorization, MVC-related visits in medical cannabis patients after 12 months was 0.33 visits per 10,000 patients; and MVC-related visits in controls was 0.21 visits per 10,000 patients - with neither change statistically significant (*p* = 0.70 and *p* = 0.60, respectively) (Table 6). However, when comparing those authorized medical cannabis to controls, MVC-related visits of − 0.11 events per 10,000 in those authorized medical cannabis (over the 12 months relative to controls) was observed which was also not statistically significant (*p* = 0.56) (Table 6). After accounting for both the immediate and temporal effects, the absolute effect of medical cannabis authorization was a non-statistcially significant increase of 4.32 events/10,000 (95%CI − 0.73 to 9.37) over the entire 12-month follow-up period. Finally, no associations were observed with respect to either MVC-related hospitalizations or emergency department visits in stratified analyses when comparing those authorized medical cannabis to controls.

**Table 5:** Cannabis motor vehicle crash healthcare utilization – one year before and one year after authorization for medical cannabis

Outcome	Cannabis Population Difference in mean number of visits/admissions per 10,000 patients from 1 year before to 1 year after medical cannabis			Matched Controls Difference in mean number of visits/admissions per 10,000 patients from 1 year prior to 1 year after index date		
	Before	After	Change	Before	After	Change
Hospitalization or Emergency Department visit as a result of motor vehicle crashes	121	95	−26	65	50	−15
Hospitalization visit as a result of motor vehicle crashes	3.3	9.8	+ 6.5	0	2.5	+ 2.5
Emergency Department visit as a result of motor vehicle crashes	118	85	−33	65	47	−18

**Table 6:** Interrupted time series analysis of healthcare utilization due to motor vehicle crash one year before and one year after authorization for medical cannabis compared to those unauthorized (*n* = 7065)

Outcome	Authorized Medical Cannabis				Unauthorized Controls				Difference				Absolute change	
	Immediate Level Change*		Temporal Trend change**		Immediate Level Change*		Temporal Trend Change**		Immediate Level Change*		Temporal Trend Change**		Combined Level and Trend Changes	
	Events/ 10,000 patients	*p*-value	Events/ 10,000 patients	*p*-value	Events/ 10,000 patients	*p*-value	Events/ 10,000 patients	*p*-value	Events/ 10,000 patients	*p*-value	Events/ 10,000 patients	*p*-value	Events/ 10,000 patients	95% Confidence intervals
Motor-vehicle related hospitalization or ED visit	4.19	0.0294	0.33	0.1731	−0.84	0.7191	0.21	0.5973	4.77	0.0034	−0.11	0.5627	4.32	−0.73 to 9.37
Motor-vehicle related hospitalization	0.040	0.9728	0.15	0.3594	0.28	0.5182	−0.0082	0.8915	−0.24	0.8662	0.19	0.6389	-1.15	-14.61 to 12.63
Motor-vehicle related ED	4.82	0.0705	0.27	0.4309	−0.84	0.7191	0.21	0.5973	3.37	0.5935	0.23	0.7575	3.92	2.65 to 5.19

*change in the month following the authorization of cannabis or the index date

**change in slope in the six months following the authorization of cannabis or the index date

*ED* Emergency department

## Discussion

This population-based study of patients authorized for medical cannabis showed an overall absolute increase (overall level and trend effects) in MVC-related visits of 2.92 per 10,000 people (compared to controls) within the first 6 months, which was largely driven by increases in MVC-related emergency department visits. However, no statistical differences in MVC-related healthcare utilization were observed in the subgroup of patients followed for up to 1 year, although the overall absolute effects were higher than the 6-month data (absolute events of 4.32 per 10,000 people). The clinical relevance of these findings at the individual level is unclear but may have important implications from a public health perspective.

The majority of previous studies of medical cannabis and MVC risk have shown inconsistent results. Certain studies report high correlation between medical cannabis/recreational cannabis use and MVC risk (Richer and Bergeron, 2009; Wright and Metts, 2016). Bonar et al. (2019) reported that DUIC behavior was higher in medical cannabis patients authorized for chronic pain than those in the general population of individuals who were reported to drive after the use of cannabis (Bonar et al., 2019). Recent Canadian reports on MVC and cannabis (Foundation, T. I. R, 2018; Alberta, 2017) indicate a general increase of fatally injured drivers who tested positive of cannabis from 2000 to 2015. Recent meta-analyses of epidemiological studies (Rogeberg and Elvik, 2016; Hartman and Huestis, 2013) including Li et al. (Li et al., 2012) also showed a significant increase of MVC risk as a result of cannabis consumption. Conversely, other meta-analyses report that the association between medical cannabis use and MVC risk is nonsignificant (Hostiuc et al., 2018) – and that only higher levels of cannabis were associated with higher MVC risk (Brubacher et al., 2019). Notably, other ITS studies (Hamilton et al., 2014) focused on recreational use and/or impairment without strictly focusing on solely medical use (Ogourtsova et al., 2018). Indeed, Masten et al. (Masten and Guenzburger, 2014) reported that medical cannabis laws may not necessarily be linked with increased MVC rates. Likewise, Neavyn et al. (Neavyn et al., 2014) reported the importance of distinguishing between medical cannabis and recreational cannabis to fully understand its effects on MVC-risk associated behavior. These discrepancies may explain the difference in outcomes associated with medical cannabis use and MVC risk among the various study populations.

The strength of our study is that it is currently the largest Canadian population-based study completed with population-based matched controls. However, our study is not without limitations. First, this is an observational study and potential spectrum bias is a concern as our cohort is based on patients who have individually sought authorization for medical cannabis. This population may not be representative of all individuals who are using cannabis for medical purposes but obtained it through other (legal or illegal) avenues.

Among the limitations, we were not able to match all the cannabis cohort patients to at least one control as noted (about 19% were not matched and were excluded from the analysis). It is unclear how this could have affected the results. This issue has probably led to an underestimation of the MVC events as the excluded patients were more likely to be older and had higher rates of morbidities. However, there is no reason to believe that the relative effects would be affected as similar characteristics would be expected in controls if matched. Although controls did not have any records of a referral to a participating cannabis clinic, it is possible these patients could have been using recreational cannabis which we could not capture. If so, this misclassification bias would have led to an underestimation of the MVC effects of cannabis in our analyses. We also have no information on patients which may have declined consent for data collection, and thus, we can make no assumptions about this group of patients or how they may have affected our results. Although patients were authorized to use medical cannabis, we cannot ensure the products were consumed as authorized by physicians or if patients elected to use alternative agents than what was authorized. Moreover, there is no method of determining if medical cannabis was in a patient’s system at the time of an MVC. Third, not all MVCs result in healthcare resource utilization and our data do not capture MVCs that did not result in injury or were less severe, thus, we only investigated major crashes resulting in healthcare utilization; not minor crashes. Lastly, we do not know whether the association may change depending on if the MVC was caused by the authorized user or someone else. As this information is from law-enforcement agencies (not available to researchers), we only focused on the user coming into the hospital/ED as a result of an MVC.

## Conclusions

Overall, this study suggests an association between medical cannabis authorization and MVC-related healthcare utilization in Ontario medical cannabis users. The clinical relevance of these findings at the individual level is unclear but may have important implications from a public health perspective. Although some may consider the risk small, a policy requiring physicians to discuss the risks of medical cannabis use while driving, should be warranted for patients who are authorized for medical cannabis. Users of medical cannabis should continue to use this medication with caution when interacting with their environments and follow all instructions concerning its use during the operation of motor vehicles.

## Data Availability

The dissemination of data results to study participants and or patient organizations in this research project is not possible/applicable. The dissemination of data results to study participants and or patient organizations in this research project is not possible/applicable as the data is de-identified. Moreover, the data is not available as only the researchers authorized by ICES have access to the data.

## Appendix

**Table 7:** Health conditions and ICD-10 codes defining the Motor-vehicle-related hospitalizations (MVC)

Condition	ICD-10
Car occupant injured in transport crash	V40*-V49*
Occupant of pick-up truck or van injured in transport crash	V50-V59
Occupant of heavy transport vehicle injured in transport crash	V60-V69

*The following fourth-character subdivisions are for use with categories V40-V48

**.0 Driver injured in nontraffic crash**

**.1 Passenger injured in nontraffic crash**

**.2 Person on outside of vehicle injured in nontraffic crash**

**.3 Unspecified car occupant injured in nontraffic crash**

**.4 Person injured while boarding or alighting**

**.5 Driver injured in traffic crash**

**.6 Passenger injured in traffic crash**

**.7 Person on outside of vehicle injured in traffic crash**

**.9 Unspecified car occupant injured in traffic crash**

Legend:

*ICD-10* International Classification of Diseases, Tenth Revision.

**Table 8:** Stratification of Authorized and Unauthorized Adult Patients by Age, Sex, Rural/Urban

Outcome	Authorized				Unauthorized				Difference			
	Immediate change*		Temporal change**		Immediate change*		Temporal change**		Immediate change*		Temporal change**	
	Events/ 10,000 patients	*p*-value	Events/ 10,000 patients	*p*-value	Events/10,000 patients	*p*-value	Events/ 10,000 patients	*p*-value	Events/ 10,000 patients	*p*-value	Events/ 10,000 patients	*P*-value
Motor-vehicle related hospitalization or ED visit	−3.15	0.3988	0.50	0.6102	−2.38	0.2879	−0.31	0.6405	−2.42	0.0138	0.889	0.0019
Age												
< 30	2.42	0.7479	−1.99	0.3082	1.45	0.7717	−2.59	0.0807	3.36	0.7622	−0.037	0.9897
31 to 60	−2.69	0.3049	1.23	0.0796	−3.57	0.1370	0.42	0.4885	0.52	0.9550	0.56	0.8143
> 60	−3.27	0.2696	−0.27	0.7182	0.47	0.9126	−0.54	0.6747	−5.09	0.6128	0.23	0.9432
Sex												
Male	−0.99	0.7140	−0.60	0.5060	−4.30	0.0419	−1.24	0.0327	18.94	0.5903	7.14	0.5987
Female	4.31	0.3496	1.67	0.2653	1.28	0.5981	1.10	0.1297	23.83	0.4015	7.43	0.7924
Urban/Rural												
Urban	−3.08	0.3364	0.087	0.9152	−2.13	0.2959	−0.19	0.7328	−5.12	0.2229	1.50	0.0403
Rural	0.71	0.9469	3.08	0.2923	1.16	0.7347	−0.39	0.6666	−6.83	0.6224	2.69	0.2782

Legend:

*ED* Emergency visi

**Table 9:** ICD codes by Case and Control in Motor-Vehicle Crashes

MVC ICD DX code	Control	Case
V405	3	3
V430	3	3
V431	0	1
V434	1	1
V435	159	141
V436	40	46
V437	1	0
V439	9	5
V445	4	14
V446	0	1
V455	1	0
V460	1	0
V465	1	0
V470	3	4
V471	1	1
V475	20	18
V476	1	2
V480	1	1
V481	0	3
V482	1	2
V483	1	0
V484	6	5
V485	11	9
V486	4	8
V489	1	2
V490	0	1
V493	0	1
V494	10	12
V495	7	4
V496	2	4
V498	2	0
V499	9	17
V505	1	0
V530	1	0
V532	0	1
V535	8	4
V536	0	3
V539	0	1
V545	0	2
V546	1	0
V575	1	1
V581	0	1
V584	6	3
V585	1	1
V586	3	1
V594	1	0
V595	0	1
V596	1	0
V599	1	0
V645	1	0
V675	1	0
V681	0	1
V684	1	2
V685	3	1
V687	1	0
V698	0	1
TOTAL	335	333

Legend**:**

*ICD* International Classification of Diseases.

**Table 10:** Matched authorized versus unmatched authorized cannabis patients

Characteristics	Authorized cannabis patients matched to a control (18662)	Authorized cannabis patients not matched to a control (***n*** = 4429)
Age		
< 21	120 (0.64)	23 (0.52)
21–30	1974 (8.55)	240 (5.42)
31–40	3606 (19.32)	547 (12.35)
41–50	3822 (20.48)	843 (19.03)
51–60	4846 (25.97)	1165 (26.30)
61–70	2858 (15.31)	878 (19.82)
71–80	1050 (5.63)	491 (11.09)
> 80	386 (2.07)	242 (5.46)
Sex (males)	10,132 (54.29)	2124 (47.96)
Rural (yes)	1798 (9.63)	519 (11.72)
Asthma	3691 (19.78)	1737 (39.22)
Musculoskeletal disorders	8256 (44.24)	3032 (68.46)
behavioural disorders	3582 (19.19)	2152 (48.59)
Cancer	1828 (9.80)	1387 (31.32)
COPD	2353 (12.61)	1706 (38.52)
Diabetes	2215 (11.87)	1518 (34.27)
Fatigue	279 (1.50)	1003 (22.65)
Metabolic disease	2609 (13.98)	2361 (53.31)
Neurlogic disorders	2892 (15.50)	2146 (48.45)
Pain	615 (3.30)	1397 (31.54)

## Abbreviations

CIHI: Canadian institutes of health research
ICES: Institute for clinical evaluative sciences
ITS: Interrupted time series
MVC: Motor vehicle crashes
SPOR: Strategy for patient-oriented research
THC: Tetrahydrocannabinol
